# Supplementary material for: Plasma membrane rather than endosomal Gq signaling drives transcriptional activity by the viral chemokine receptor US28 in glioblastoma
Source: bioRxiv. 2025 Jun 15:2025.06.12.659276. Preprint. [Version 1] doi: 10.1101/2025.06.12.659276 (PMC12259172; doi:10.1101/2025.06.12.659276)
Supplement: Supplement 1 [file media-1.pdf]

SUPPLEMENTARY INFORMATION

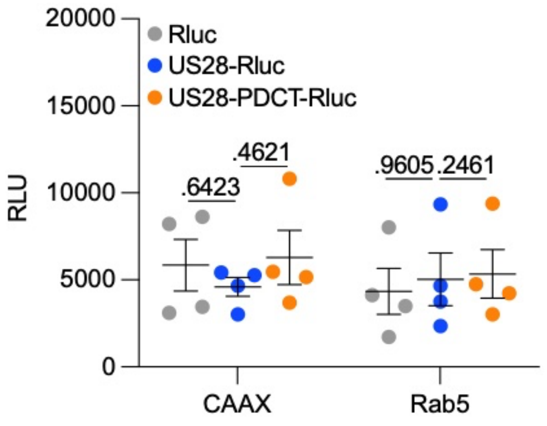

Supplementary Fig. 1: Similar total expression of US28-Rluc, US28-PDCT-Rluc, or Rluc alone across U251 cells transfected transiently to evaluate the subcellular expression of these constructs at the plasma membrane (CAAX) or early endosomes (Rab5). Data represent the luminescence expressed as relative luminescence units (RLU) monitored in U251 cells transiently transfected with US28-Rluc, US28-PDCT-Rluc or Rluc alone. Data are shown as means ± SEM of *n* = 4 independent biological replicates performed in technical quadruplicates and were analyzed by two-way ANOVA with Sidak's post hoc test.

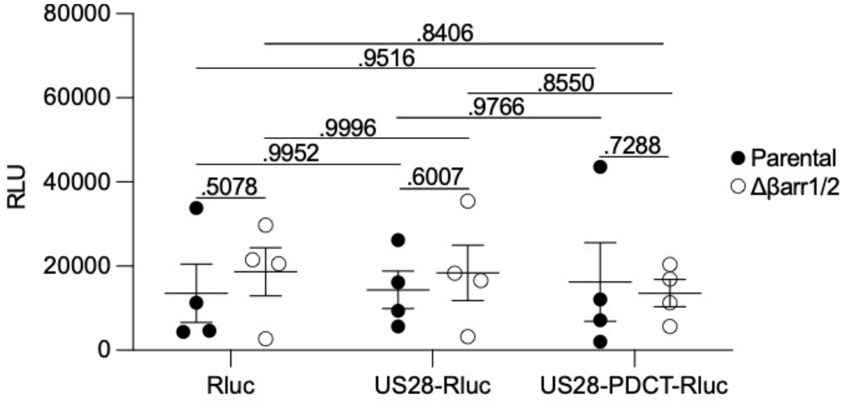

Supplementary Fig. 2: Similar total expression of US28-Rluc, US28-PDCT-Rluc, or Rluc alone across parental and β-arrestin-deficient HEK-293 cells transfected transiently to evaluate the recruitment of mGq-GFP to the Rluc-fused constructs. Data represent the luminescence expressed as relative luminescence units (RLU) monitored from parental and β-arrestin-deficient HEK-293 cells transiently transfected with US28-Rluc, US28-PDCT-Rluc or Rluc alone. Data are shown as means ± SEM of n = 4 independent biological replicates performed in technical quadruplicates and were analyzed by two-way ANOVA with Tukey's post hoc test.

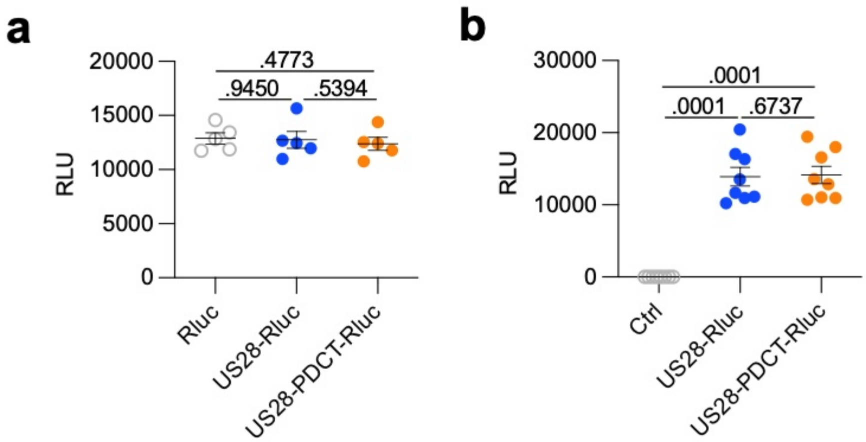

Supplementary Fig. 3 : Similar total expression of US28-Rluc, US28-PDCT-Rluc, or Rluc alone across U251 cells transfected transiently to evaluate the constitutive production of second messengers. a Luminescence expressed as relative luminescence units (RLU) monitored in U251 cells transiently transfected with US28-Rluc, US28-PDCT-Rluc, or Rluc alone used to monitor the constitutive DAG production. Data are shown as means ± SEM of n = 5 independent biological replicates performed in technical quadruplicates. **b** RLU monitored from U251 cells transiently transfected with US28-Rluc, US28-PDCT-Rluc, or vector alone used to monitor the constitutive IP_1 production. Data are shown as means ± SEM of n = 8 independent biological replicates performed in technical quadruplicates. Indicated p values are derived from one-way ANOVA with Tukey’s post hoc test.

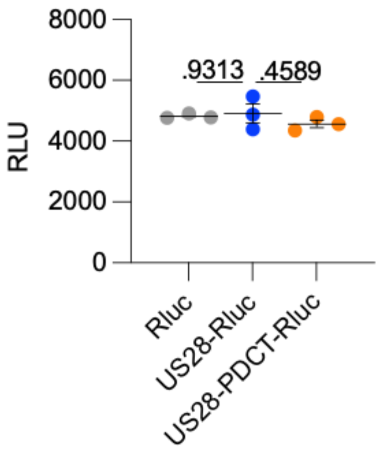

Supplementary Fig. 4: Similar total expression of US28-Rluc, US28-PDCT-Rluc, or Rluc alone across U251 cells transfected transiently for RNA sequencing. Luminescence expressed as relative luminescence units (RLU) monitored in U251 cells transiently transfected with US28-Rluc, US28-PDCT-Rluc, or Rluc used for RNA sequencing. Data are shown as means ± SEM of n = 3 independent biological replicates performed in technical triplicates or duplicates. One-way ANOVA with Dunnett's post hoc test.

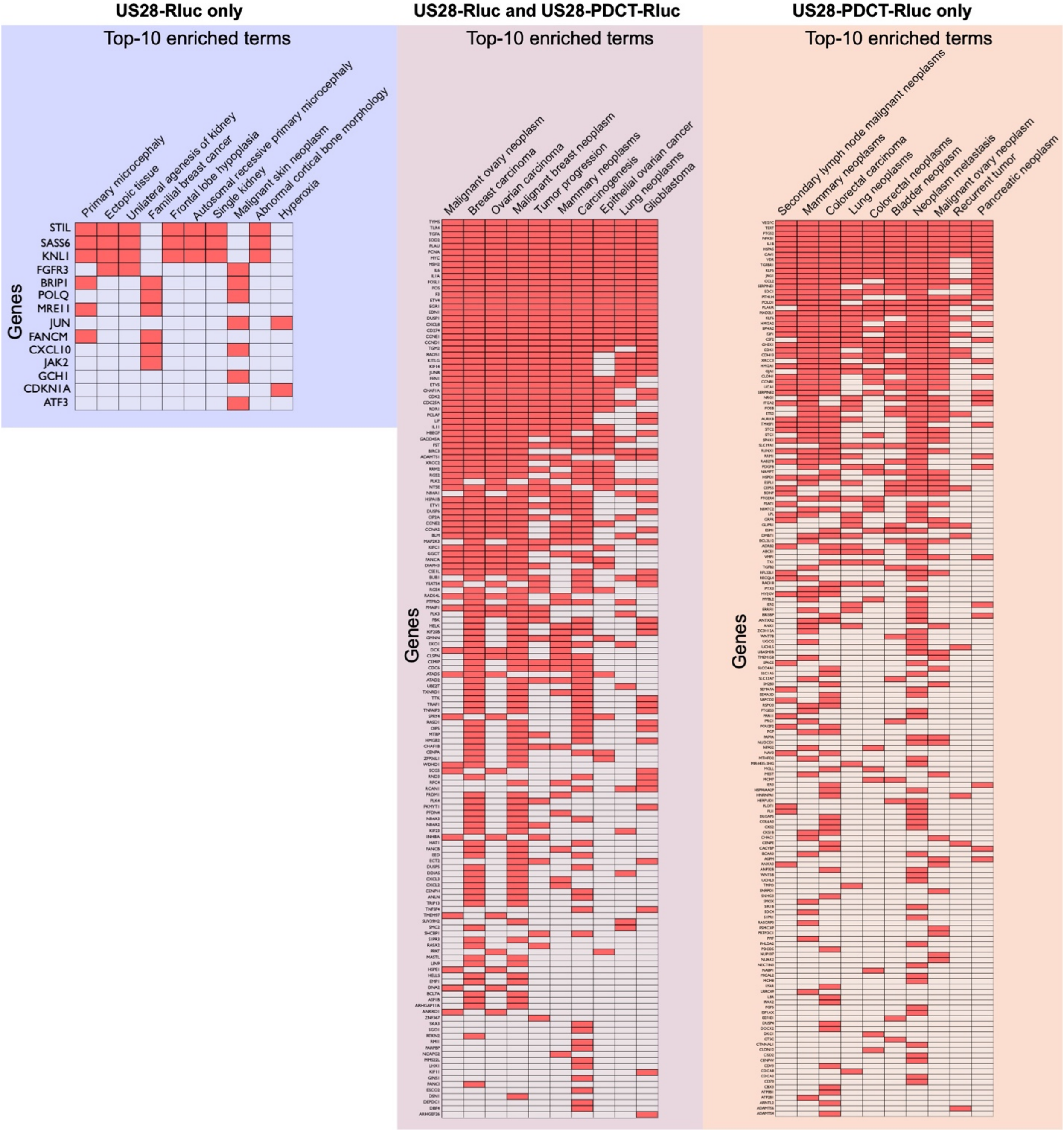

Supplementary Fig. 5: Clustergram showing the top 10 enriched terms in the DisGeNET library with the associated input genes. From the 83 genes only upregulated by US28-Rluc, the 247 genes upregulated by both US28-Rluc and US28-PDCT-Rluc, or the 347 genes upregulated by US28-PDCT-Rluc only.

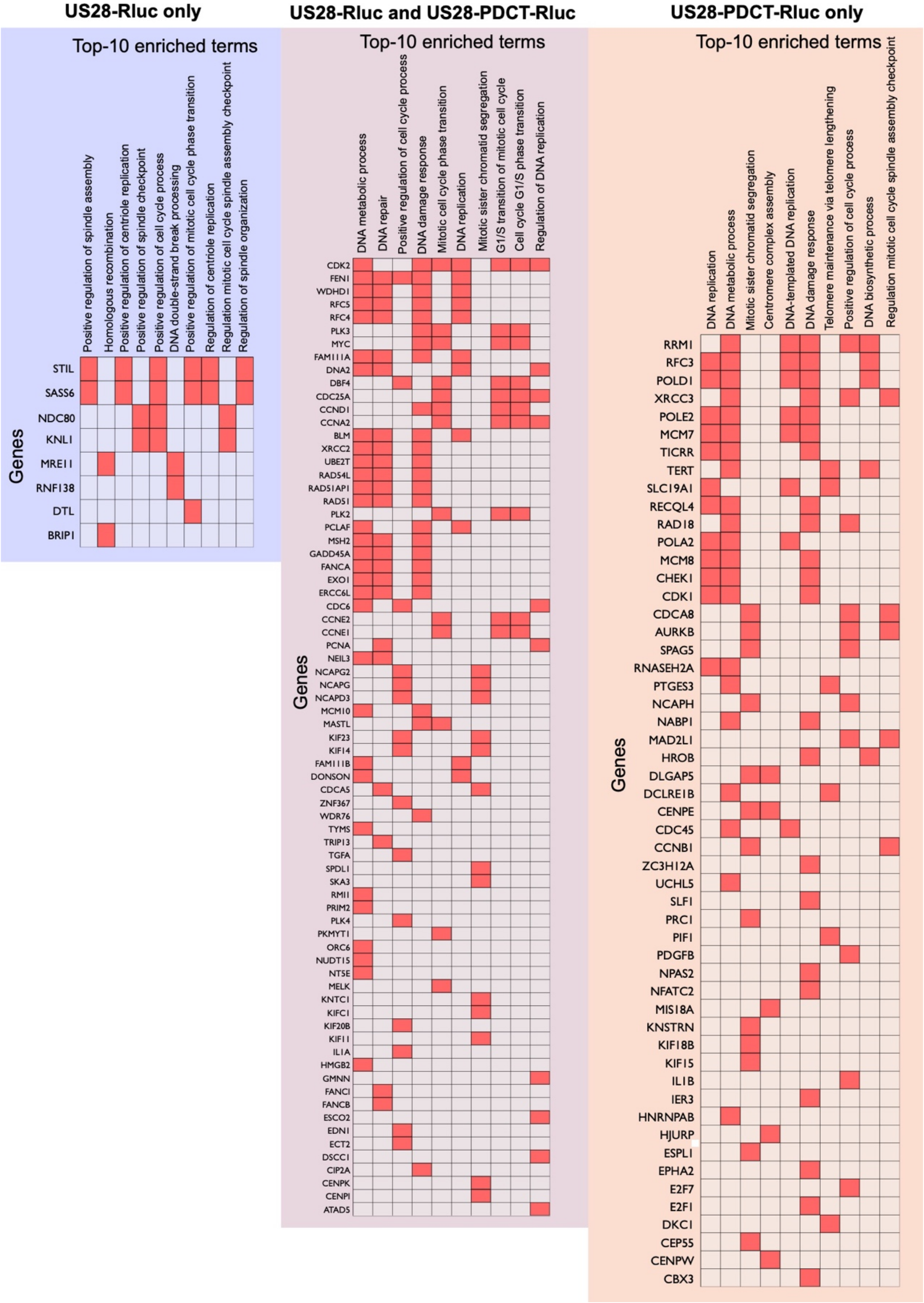

Supplementary Fig. 6: Clustergram showing the top 10 enriched terms in the GO Biological Process 2025 library with the associated input genes. From the 83 genes only upregulated by US28-Rluc, the 247 genes upregulated by both US28-Rluc and US28-PDCT-Rluc, or the 347 genes upregulated by US28-PDCT-Rluc only.

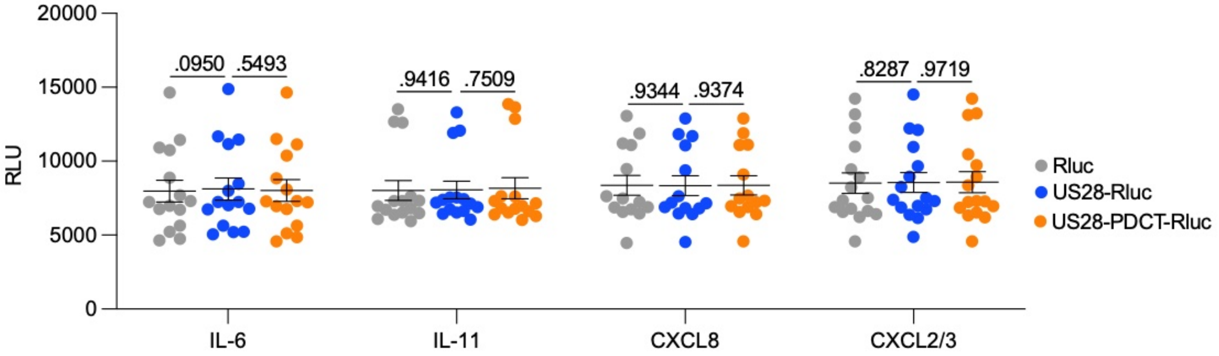

Supplementary Fig. 7: Similar total expression of US28-Rluc, US28-PDCT-Rluc, or Rluc alone across U251 cells transfected transiently for cytokine secretion measurement. RLU monitored in U251 cells transiently transfected with US28-Rluc, US28-PDCT-Rluc, or Rluc alone used to monitor the production of cytokines in the conditioned media. Data are shown as means ± SEM (*n* = 15 for IL-6 and IL-11, *n* = 14 for CXCL8, *n* = 16 for CXCL2/3) performed in technical quadruplicates. Two-way ANOVA with Sidak's post hoc test.

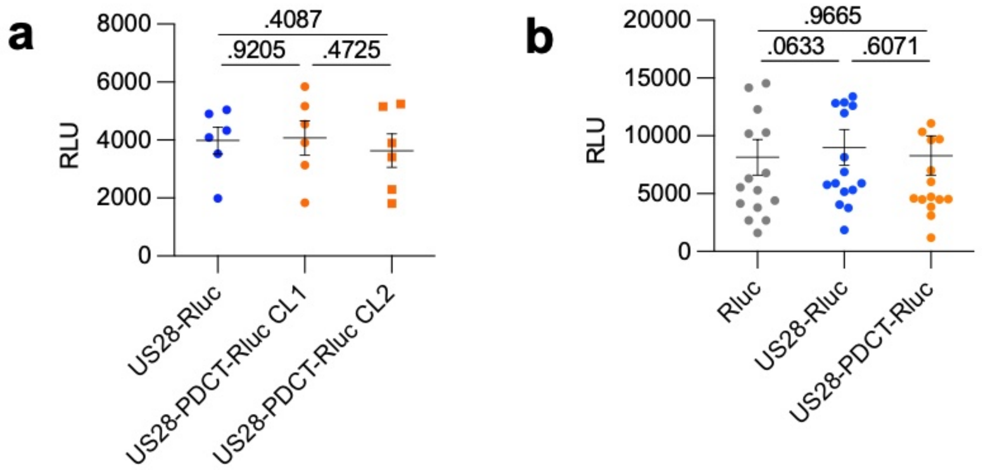

Supplementary Fig. 8: Similar total expression of US28-Rluc, US28-PDCT-Rluc, or Rluc alone across U251 cells used for generation of spheroids or neutrophil migration assay. **a** Luminescence expressed as relative luminescence units (RLU) monitored in clonal U251 cells stably expressing US28-Rluc, or US28-PDCT-Rluc used to generate spheroids. Data are shown as means ± SEM of *n* = 6 independent biological replicates performed in technical quadruplicates. **b** RLU monitored from U251 cells transiently transfected with US28-Rluc, US28-PDCT-Rluc, or Rluc alone for which the conditioned media was used to monitor neutrophil migration. Data are shown as means ± SEM (*n* = 16) performed in technical quadruplicates. **a,b:** one-way ANOVA with Tukey's post hoc test.

Supplementary Table 1: RNA sequencing – raw data. Raw data from FASTQ files from three independent biological replicates of RNA samples extracted from U251 cells expressing US28-Rluc, US28-PDCT-Rluc, or Rluc alone.

Supplementary Table 2: Differential gene expression analysis (DGEA) – US28-Rluc versus Rluc. DGEA from three independent biological replicates of RNA samples extracted from U251 cells expressing US28-Rluc or Rluc.

Supplementary Table 3: Differential gene expression analysis (DGEA) – US28-PDCT-Rluc versus Rluc. DGEA from three independent biological replicates of RNA samples extracted from U251 cells expressing US28-PDCT-Rluc or Rluc.

Supplementary Table 4: List of genes upregulated by US28-Rluc only, US28-PDCT-Rluc only, or both. From DGEA from three independent biological replicates of RNA samples extracted from U251 cells expressing US28-Rluc, US28-PDCT-Rluc, or Rluc.
